# Representativeness of whole-genome sequencing approaches in England: the importance for understanding inequalities associated with SARS-CoV-2 infection

**DOI:** 10.1017/S0950268823001541

**Published:** 2023-09-20

**Authors:** Katherine A. Twohig, Katie Harman, Asad Zaidi, Shirin Aliabadi, Sophie G. Nash, Mary Sinnathamby, Ian Harrison, Eileen Gallagher, Natalie Groves, Frank Schwach, Clare Pearson, Alicia Thornton, Richard Myers, Meera Chand, Simon Thelwall, Gavin Dabrera

**Affiliations:** 1 COVID-19 National Epidemiology Cell, UKHSA, London, UK; 2 COVID-19 Vaccines and Epidemiology Division, Public Health Programmes, Clinical and Public Health Group, UKHSA, London, UK; 3 Pathogen Genomics, Science Group, UKHSA, London, UK; 4 TARZET Division, Clinical and Emerging Infections Directorate, Clinical and Public Health Group, UKHSA, London, UK

**Keywords:** COVID-19, deprivation, socio-economic status, ethnicity, travel, SARS-CoV-2, sequencing, variants, England, inequalities

## Abstract

Whole-genome sequencing (WGS) information has played a crucial role in the SARS-CoV-2 (COVID-19) pandemic by providing evidence about variants to inform public health policy. The purpose of this study was to assess the representativeness of sequenced cases compared with all COVID-19 cases in England, between March 2020 and August 2021, by demographic and socio-economic characteristics, to evaluate the representativeness and utility of these data in epidemiological analyses. To achieve this, polymerase chain reaction (PCR)-confirmed COVID-19 cases were extracted from the national laboratory system and linked with WGS data. During the study period, over 10% of COVID-19 cases in England had WGS data available for epidemiological analysis. With sequencing capacity increasing throughout the period, sequencing representativeness compared to all reported COVID-19 cases increased over time, allowing for valuable epidemiological analyses using demographic and socio-economic characteristics, particularly during periods with emerging novel SARS-CoV-2 variants. This study demonstrates the comprehensiveness of England’s sequencing throughout the COVID-19 pandemic, rapidly detecting variants of concern, and enabling representative epidemiological analyses to inform policy.

## Introduction

SARS-CoV-2 has demonstrated itself to be a rapidly mutating virus, which has highlighted the value of large-scale, accessible, and timely genomic surveillance [1]. The COVID-19 pandemic has been the first pandemic where genomic technology has been widely available at such scale [2]. In the United Kingdom (UK), the COVID-19 Genomics UK Consortium (COG-UK) was established in April 2020 to provide large-scale, rapid whole-genome sequencing (WGS) for SARS-CoV-2 [3]. The UK has been at the forefront of global sequencing throughout the pandemic, with over two million genomes sequenced by February 2022 [4].

WGS has significantly contributed to the understanding of genomic diversity and evolution of the SARS-CoV-2 virus, as well as clinical and epidemiological characteristics of the infection. Through the rapid identification of novel variants, sequencing has been crucial in providing evidence to inform the implementation of public health policies, such as those that were established to manage the response to the alpha (B.1.1.7) variant and other lineages in early 2021 [5, 6]. To maximise population-level epidemiological insights, WGS must be as representative of the population as possible. Representativeness is beneficial both to ensure analyses are unbiased and to aid in global sharing of sequences, as per the World Health Organization recommendations [7].

It has been evidenced throughout the pandemic that COVID-19 has had varying impacts on population sub-groups, with older age, black, Asian, and minority ethnicity (BAME), and residence in areas of greater socio-economic deprivation, indicating greater risk of infection, as well as more severe outcomes including death [8, 9]. The capability and capacity to monitor health inequalities associated with emerging SARS-CoV-2 variants are essential to inform public health policy, but they rely on sequencing of cases providing sufficient information on groups in relation to key characteristics. It is therefore critical to understand the representativeness of sequenced cases in relation to confirmed cases overall.

We evaluated representativeness by assessing the proportion of sequenced COVID-19 cases in England, including changes over time, and by key demographic characteristics including sex, age, geography, indices of deprivation (IMD), ethnicity, and travel status.

## Methods

Polymerase chain reaction (PCR)-confirmed COVID-19 cases in England reported to the national laboratory system, Second Generation Surveillance System (SGSS) [10], with specimen dates between 1 March 2020 and 31 August 2021, were linked with sequencing data uploaded to the Cloud Infrastructure for Big Data Microbial Bioinformatics (CLIMB) [11]. This linkage was based on specimen identifiers assigned at the diagnostic sites and submitted to Public Health England (PHE) either through secure file transfer or uploaded to CLIMB [12]. Linkage between patient data and sequencing results was performed securely within the PHE environment. Records were only linked if the sequence passed quality assurance thresholds, deeming it suitable for genomic analysis.

Key attributes of the case and test results were extracted from SGSS, including sex, age, geography of residence, and IMD, where quintile 1 represents the most deprived and 5 represents the least deprived [13], ethnicity, and reporting pillar of the first positive test. The test pillar represented the laboratory and reporting pathway of the positive result. Pillar 1 (P1) includes tests undertaken by public health, National Health Service (NHS), and privately contracted laboratories, and some targeted testing such as people in hospital and workplace screening. Pillar 2 (P2) tests were generally community-based and were reported into SGSS through NHS Digital Platforms. Information about recent international travel was also assessed, which was defined as arrival from outside of the UK within 14 days before the positive test date. This was derived from the linkage of five sources: arrival forms from recent travellers, contact tracing information, travel information included on test request forms, reports from the international arrival testing programme and questionnaires submitted from regional health protection teams.

Overall, the proportion of sequenced cases was assessed by demographic and epidemiological characteristics, with a focus on three time periods: (A) March to July 2020, (B) August 2020 to April 2021, and (C) May to August 2021. These intervals were calculated based on the specimen collection dates and included key changes in the epidemiology, reporting, or sequencing capacity of COVID-19 in England. Period A reflected the initial epidemic, after the first sporadic cases, and the introduction of the P2 testing pathway. Period B started from the beginning of the second wave of cases in England and through the winter months when sequencing capacity was further increased, particularly for P2 [14]. Period C began in the spring of 2021 following the winter peak and included the transition to greater sequencing capacity being taken over by public health laboratories instead of academic sequencing partners. The proportions of cases in each period and category were calculated for both total cases and sequenced cases, and then, a ratio of these proportions was calculated. Chi-squared tests were conducted to compare whether the distributions of characteristics were similar between total cases and sequenced cases (*P* < 0.001).

## Results

**Figure 1.**
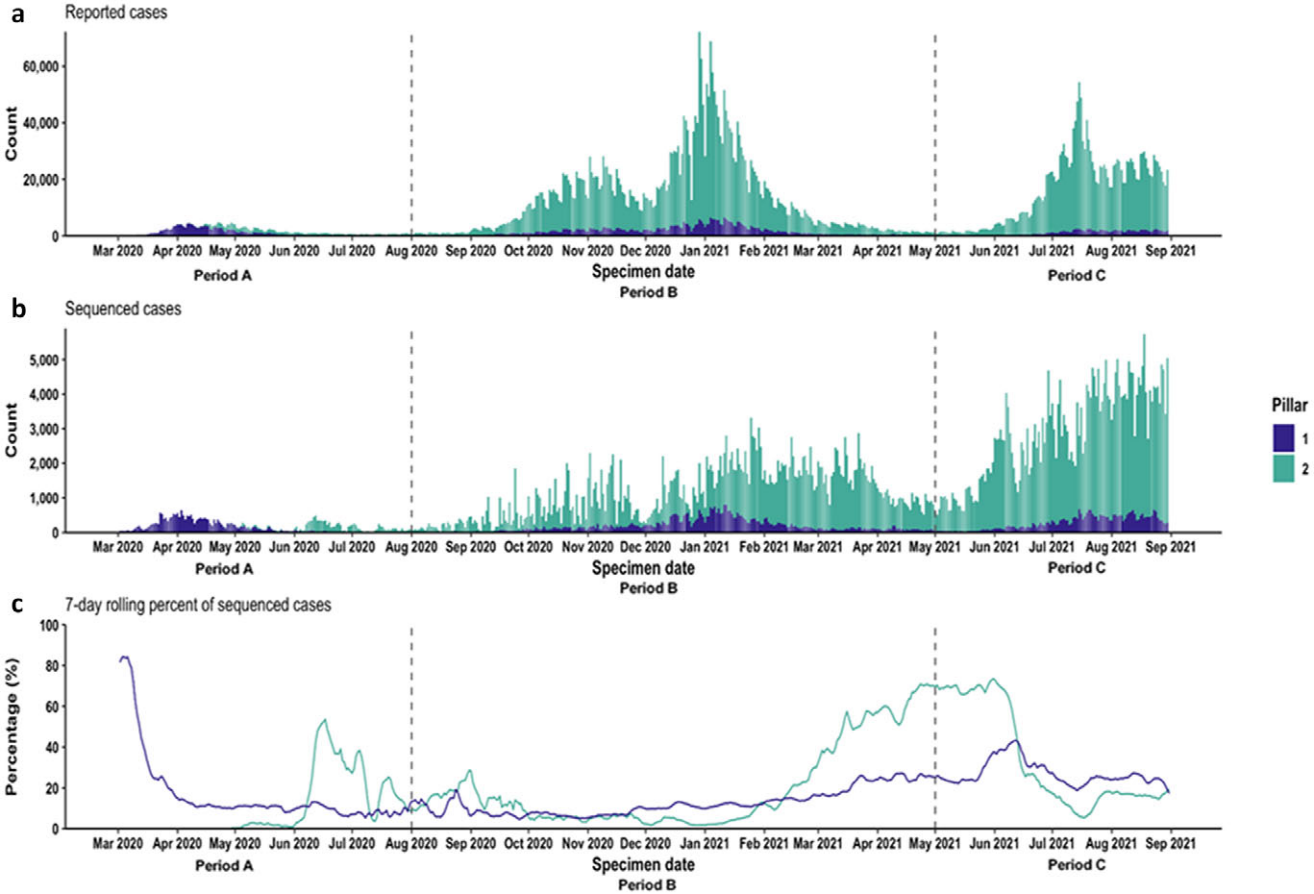
(a) PCR-confirmed COVID-19 cases reported in England, by pillar. (b) PCR-confirmed COVID-19 cases linked with quality-assessed whole-genome sequencing results from CLIMB. (c) 7-day rolling per cent of cases that were sequenced.

Between 01 March 2020 and 31 August 2021, there were 5,810,945 PCR-confirmed COVID-19 cases reported in England, of which 688,203 (11.8%) were linked with quality-assessed WGS results from CLIMB. Through all three time periods, P1 had a lower testing volume than P2, yet the proportion of linked sequences varied over time and by testing pillar (Figure 1). The highest proportion of P1 cases linked to sequences occurred when case numbers were very low at the beginning of the pandemic, with a slow increase to a further peak in June 2021. During periods with higher case numbers, P1 sequencing volumes increased, as shown by the similar proportions of sequenced cases in April 2020, January 2021, and August 2021.

At the start of the study period (Period A), when cases were less than 100 per day in March 2020, approximately 80% of cases were sequenced (Figure 1). P2 testing began in mid-April 2020, and sequencing for P2 tests started in June 2020. The proportion of P2 cases sequenced was highest when case numbers were low, particularly in June 2020, when the proportion occasionally exceeded 50%, and, in the spring of 2021, when the highest proportion throughout the study was observed at 73.5% in May 2021 (Figure 1).

From August 2020 (Period B), case numbers began to rise, particularly in P2, which had increased testing capacity. Although the absolute number of sequenced cases increased, they reflected a smaller proportion of the overall cases. P1 sequencing volumes peaked in the middle of January 2021 at 12.8%, whereas P2 sequencing peaked at 13.0% in the end of January 2021. The proportion sequenced then rose again in line with decreases in case numbers, with P1 reaching a peak of 43.3% sequenced in mid-June 2021, and P2 reaching 73.5% at the end of May. During Period C, P2 sequencing capacity greatly increased, and the proportion of cases sequenced remained high following this rise in capacity. In August 2021, an average of 24.3% of P1 cases were sequenced, compared with 16.8% for P2.

Overall, the ratio of the proportion of sequenced cases to the proportion of total cases shows that sequencing results were broadly representative of the underlying case populations (Table 1); however, there were some over-represented groups. For all time periods, there were a higher proportion of P1 cases that were sequenced, compared with the proportion of P1 among total cases.

**Table 1.** Demographic and epidemiological characteristic breakdowns of overall testing and sequencing, 01 March 2020 to 31 August 2021

Category	Period A 01 March 2020–31 July 2020				Period B 01 August 2020–30 April 2021				Period C 01 May 2021–31 August 2021			
	Sequenced cases	Total cases	Ratio^a^	X^2^^b^	Sequenced cases	Total cases	Ratio^a^	X^2^^b^	Sequenced cases	Total cases	Ratio^a^	X^2^^b^
Pillar 1	21444 (70.0%)	164422 (62.4%)	1.1	^b^	47219 (15.5%)	430379 (12.1%)	1.3	^b^	33610 (9.5%)	137061 (6.9%)	1.4	^b^
Pillar 2	9170 (30.0%)	99131 (37.6%)	0.8		257599 (84.5%)	3126996 (87.9%)	1.0		319161 (90.5%)	1852956 (93.1%)	1.0	
												
Male	14743 (48.2%)	113113 (42.9%)	1.1	^b^	147038 (48.2%)	1652766 (46.5%)	1.0	^b^	176806 (50.1%)	998562 (50.2%)	1.0	^b^
Female	15732 (51.4%)	147157 (55.8%)	0.9		156776 (51.4%)	1879585 (52.8%)	1.0		174723 (49.5%)	981855 (49.3%)	1.0	
Unknown	139 (0.5%)	3283 (1.2%)	0.4		1004 (0.3%)	25024 (0.7%)	0.4		1242 (0.4%)	9600 (0.5%)	0.8	
												
<10	548 (1.8%)	3609 (1.4%)	1.3	^b^	16784 (5.5%)	174232 (4.9%)	1.1	^b^	22318 (6.3%)	134020 (6.7%)	0.9	^b^
10–19	1004 (3.3%)	6806 (2.6%)	1.3		34730 (11.4%)	386782 (10.9%)	1.0		75183 (21.3%)	424304 (21.3%)	1.0	
20–29	3649 (11.9%)	31045 (11.8%)	1.0		56007 (18.4%)	669580 (18.8%)	1.0		91595 (26.0%)	519725 (26.1%)	1.0	
30–39	3920 (12.8%)	35374 (13.4%)	1.0		58780 (19.3%)	647157 (18.2%)	1.1		59662 (16.9%)	339529 (17.1%)	1.0	
40–49	3993 (13.0%)	37010 (14.0%)	0.9		48195 (15.8%)	558089 (15.7%)	1.0		43793 (12.4%)	242852 (12.2%)	1.0	
50–59	4421 (14.4%)	41955 (15.9%)	0.9		42802 (14.0%)	527237 (14.8%)	0.9		32548 (9.2%)	181966 (9.1%)	1.0	
60–69	3113 (10.2%)	26509 (10.1%)	1.0		22612 (7.4%)	277445 (7.8%)	0.9		15225 (4.3%)	84426 (4.2%)	1.0	
70–79	3469 (11.3%)	26535 (10.1%)	1.1		11787 (3.9%)	146175 (4.1%)	1.0		7876 (2.2%)	41624 (2.1%)	1.0	
80+	6477 (21.2%)	54349 (20.6%)	1.0		12875 (4.2%)	162447 (4.6%)	0.9		4510 (1.3%)	20381 (1.0%)	1.3	
Unknown	20 (0.1%)	361 (0.1%)	1.0		246 (0.1%)	8231 (0.2%)	0.5		61 (0.0%)	1190 (0.1%)	0.0	
												
East Midlands	3621 (11.8%)	24023 (9.1%)	1.3	^b^	20703 (6.8%)	303372 (8.5%)	0.8	^b^	26322 (7.5%)	169339 (8.5%)	0.9	^b^
East of England	6630 (21.7%)	27012 (10.2%)	2.1		27421 (9.0%)	381203 (10.7%)	0.8		34430 (9.8%)	181858 (9.1%)	1.1	
London	3644 (11.9%)	38542 (14.6%)	0.8		57079 (18.7%)	687168 (19.3%)	1.0		53684 (15.2%)	274960 (13.8%)	1.1	
North East	1562 (5.1%)	15471 (5.9%)	0.9		22237 (7.3%)	176835 (5.0%)	1.5		18833 (5.3%)	139000 (7.0%)	0.8	
North West	4986 (16.3%)	48406 (18.4%)	0.9		61439 (20.2%)	555010 (15.6%)	1.3		66049 (18.7%)	329600 (16.6%)	1.1	
South East	1659 (5.4%)	35915 (13.6%)	0.4		31409 (10.3%)	484155 (13.6%)	0.8		44515 (12.6%)	253350 (12.7%)	1.0	
South West	2324 (7.6%)	13625 (5.2%)	1.5		12454 (4.1%)	205975 (5.8%)	0.7		40251 (11.4%)	199935 (10.0%)	1.1	
West Midlands	1252 (4.1%)	27881 (10.6%)	0.4		26517 (8.7%)	399368 (11.2%)	0.8		30034 (8.5%)	203356 (10.2%)	0.8	
Yorkshire and Humber	4935 (16.1%)	32539 (12.3%)	1.3		45441 (14.9%)	359049 (10.1%)	1.5		38439 (10.9%)	236166 (11.9%)	0.9	
Unknown	1 (0.0%)	139 (0.1%)	0.0		118 (0.0%)	5240 (0.1%)	0.0		214 (0.1%)	2453 (0.1%)	1.0	
												
IMD-1 (most deprived)	8457 (27.6%)	66481 (25.2%)	1.1	^b^	85863 (28.2%)	863217 (24.3%)	1.2	^b^	79868 (22.6%)	452926 (22.8%)	1.0	^b^
IMD-2	6360 (20.8%)	58838 (22.3%)	0.9		70161 (23.0%)	809832 (22.8%)	1.0		73800 (20.9%)	413522 (20.8%)	1.0	
IMD-3	6029 (19.7%)	51089 (19.4%)	1.0		57139 (18.7%)	695616 (19.6%)	1.0		68859 (19.5%)	385127 (19.4%)	1.0	
IMD-4	5266 (17.2%)	47212 (17.9%)	1.0		50257 (16.5%)	631373 (17.7%)	0.9		66153 (18.8%)	374042 (18.8%)	1.0	
IMD-5 (least deprived)	4501 (14.7%)	39794 (15.1%)	1.0		41280 (13.5%)	552097 (15.5%)	0.9		63877 (18.1%)	361947 (18.2%)	1.0	
Unknown	1 (0.0%)	139 (0.1%)	0.0		118 (0.0%)	5240 (0.1%)	0.0		214 (0.1%)	2453 (0.1%)	1.0	
												
Asian	5412 (17.7%)	33983 (12.9%)	1.4	^b^	44457 (14.6%)	505222 (14.2%)	1.0	^b^	33904 (9.6%)	153467 (7.7%)	1.2	^b^
Black	1356 (4.4%)	12238 (4.6%)	1.0		12677 (4.2%)	161450 (4.5%)	0.9		13872 (3.9%)	72778 (3.7%)	1.1	
Mixed	415 (1.4%)	3245 (1.2%)	1.2		6677 (2.2%)	81548 (2.3%)	1.0		10459 (3.0%)	59612 (3.0%)	1.0	
Other	469 (1.5%)	3832 (1.5%)	1.0		6135 (2.0%)	67260 (1.9%)	1.1		5321 (1.5%)	25584 (1.3%)	1.2	
White	20599 (67.3%)	178911 (67.9%)	1.0		222457 (73.0%)	2598885 (73.1%)	1.0		276763 (78.5%)	1609735 (80.9%)	1.0	
Unknown	2363 (7.7%)	31344 (11.9%)	0.6		12415 (4.1%)	143010 (4.0%)	1.0		12452 (3.5%)	68841 (3.5%)	1.0	
												
International Traveller					5354 (1.8%)	18054 (0.5%)	3.6	^b^	11585 (3.3%)	46046 (2.3%)	1.4	^b^
Unknown travel	30614 (100.0%)	263553 (100.0%)	1.0		299464 (98.2%)	3539321 (99.5%)	1.0		341186 (96.7%)	1943971 (97.7%)	1.0	

a Ratio is defined as the proportion of total cases to the proportion of sequenced cases.

b *P*-values <0.001, obtained using chi-squared tests for the stratum.

Throughout all time periods, sequenced cases were broadly representative of total cases by age group and sex, with the ratio of total to sequenced cases being close to one throughout, aside from Period A, which saw slightly larger proportions of younger age groups sequenced.

There was strong statistical evidence that the proportion of cases that were sequenced varied by geography (*P* < 0.001). The proportion of cases that were sequenced was highest in the East of England (21.7%) and lowest in the West Midlands (4.1%) in Period A, with the ratio of total to sequenced cases being 2.1 and 0.4, respectively. In Period B, the proportion sequenced was highest in the North West (20.2%) and lowest in the South West (4.1%), with ratios of 1.3 and 0.7, respectively. In Period C, there was the least amount of geographic variation, with a range of ratios from 0.8 (lowest proportion sequenced in the North East with 5.3%) and 1.1 (highest sequenced in the North West with 18.7%).

By IMD quintile, the proportion of cases that were sequenced was highest in the most deprived quintile, IMD-1, (27.6%) and lowest in the least deprived IMD-5 (14.7%) in Period A. The decreasing trend in proportions sequenced across IMD quintiles remained through Periods B and C, with the least differences observed in Period C ranging from IMD-1 (22.5%) to IMD-5 (18.1%).

Those of Asian ethnicity were more highly represented in sequenced cases during Period A, with a ratio of total to sequenced cases of 1.4, followed by those of mixed ethnicity with a ratio of 1.2. There were no major disproportionalities in Period B, but again in period C those of Asian ethnicity were more highly represented with a ratio of 1.2.

Travel data were not available for Period A, and the proportion of all cases that were travel-related during Periods B and C was 0.5% and 2.3% of all cases, respectively (Table 1). For the two periods when travel data were available, there were a higher proportion of travel-related cases that were sequenced compared with the proportion of all cases that were travel-related, with ratios of 3.6 and 1.4, respectively.

## Discussion

More than 10% of all PCR-confirmed COVID-19 cases in England between March 2020 and August 2021 had WGS data available for national epidemiological analysis. As a novel pathogen with initially few cases, most of the early SARS-CoV-2 samples were sequenced to gain early insights into the virus. The proportion of sequenced cases was inversely related to the number of cases per day, with the highest proportions sequenced during periods of lower prevalence, likely reflecting sequencing capacity within the laboratory. As the pandemic progressed and sequencing capacity increased, sequenced cases more closely reflected the demographics of total cases and comprised more P2 (community) samples, which captured population-level assessment of the emergence and spread of new variants, providing useful information on variant prevalence. There were some sequencing disproportionalities by geographic location, likely a result of variation in sampling methodology, and operational or logistical considerations. However, the overall disproportionality between regions decreased over time from periods A to C. Evidence of targeted sequencing was also observed, including an emphasis on international travellers and P1 testing.

Sequencing was broadly representative of total cases when broken down by age group. However, variations were observed in ethnicity and deprivation, which were key health inequalities highlighted in the pandemic [9]. In particular, cases of Asian ethnicity were more likely to be sequenced than people of other ethnicities in Periods A and C and those of mixed ethnicity were more highly represented in Period A. Higher representativeness of different ethnic groups during certain periods was important in developing insights for minority at-risk population groups, including related inequalities, as demonstrated by analysis during the emergence of the delta variant [15]. Overall, the moderate over-representation of some ethnic groups in the study findings informs our understanding of the inequitable impact of COVID-19 in specific communities, but further explanatory work, such as analysis of hospitalisation data, would be needed to reduce any impact of selection bias and more comprehensively assess disproportionate burden.

Throughout the pandemic, there has been a higher burden of disease in more deprived residential areas [15]. Early sequencing coverage of cases from more deprived geographies in this study provides further insight into these trends, particularly in relation to the emergence of new variants. Overall, the representativeness of sequencing improved over time. During Period A, while the proportion of cases with sequencing data were broadly similar, those residing in more deprived areas (quintile 1) were more likely to be sequenced, which persisted during Period B despite the significant increase in sequencing capacity. By Period C, proportions sequenced aligned to be generally representative of the total cases in each IMD quintile. A higher proportion of sequenced cases from persons of Asian ethnicity and those from more deprived areas suggest that ethnicity and deprivation may have intersected with other characteristics that affected sample selection, such as more severe infections being hospitalised or targeted geographic sequencing related to emerging variants [16]. However, due to incomplete information on why people sought testing and which route they may have accessed for their initial positive test (P1 or P2), we cannot fully assess the relationship between severity and sample selection.

Some of the present study findings, particularly around the testing pillar, reflect the establishment and expansion of WGS. The testing pillar determined the journey of a specimen and affected the probability of being selected for sequencing. Examples of this include Period A, when a disproportionately greater number of cases in East of England were sequenced. This may have been an artefact reflecting the geographical bases of laboratories and the increasing sequencing capacity being established at this time. This was also observed in Period B, when hospitalisation rates were high in the northern regions and the sequencing through P1 was over-represented [17]. Later, fluctuations in geographical representativeness may also reflect periods when there was surge testing in certain geographical areas, increasing case ascertainment [18].

Overall, national co-ordination of sequencing sample selection was successful in ensuring that it was representative of COVID-19 cases in England. However, there may have been some bias introduced when different sampling strategies were employed. During the early stages of the pandemic, despite high proportions of cases being sequenced, access to PCR testing was limited and therefore confirmed cases would not have been representative of the true burden of infection in the population. Another data limitation is that pillar assignment reflects the testing route of an individual’s earliest positive specimen; therefore, in instances where someone tested positive across both pillars and their subsequent test was sequenced, the pillar assignment in this analysis would not match the sequenced result. Lastly, the representativeness of overall cases might have been affected by the use of lateral flow tests (LFTs), which were distributed to local authorities in November 2020 [19] and were available to the general public from April 2021 [20]. Despite guidance that positive LFTs should be followed by confirmatory PCR testing through the majority of the study period [21], this was not always followed and WGS is not possible without PCR samples. Data on the number of tests sent for sequencing and subsequent failure rate were not available, and differences in sequencing attempts by demographic group are not known.

This study is greatly strengthened by access to high-quality data on COVID-19 cases and consequent sequencing, allowing for an in-depth nationwide analysis. This analysis demonstrates that sequenced cases of COVID-19 between March 2020 and August 2021 in England were generally representative of all cases by key socio-economic characteristics, such as the most deprived who were disproportionately affected by the pandemic, providing important utility for these sequencing data. Future work will be needed to explore sequencing trends in later time periods and to consider the effects on representativeness from changes to SARS-CoV-2 testing approaches in England [22], particularly in relation to deprivation and ethnicity. This study has provided evidence to support the use of sequencing data for rigorous epidemiological analyses, including, but not limited to, assessing variant severity, vaccine effectiveness, and household transmission, which have been crucial in understanding health inequalities and informing the national COVID-19 pandemic response [23–27].

## Data Availability

The individual-level nature of the data used risk individuals being identified, or being able to self-identify, if the data are released publicly. Requests for access to these non-publicly available data should be directed to UKHSA.
